# Association between *SULT1A1 Arg213His* (rs9282861) Polymorphism and Risk of Breast Cancer: a Systematic Review and Meta-Analysis

**Published:** 2017-10-14

**Authors:** Mohammad Forat-Yazdi, Mohammadali Jafari, Saeed Kargar, Seyed Mojtaba Abolbaghaei, Rezvan Nasiri, Soudabeh Farahnak, Elnaz Foroughi, Hossein Neamatzadeh

**Affiliations:** ^1^ Department of Internal Medicine, Shahid Sadoughi University of Medical Sciences, Yazd, Iran; ^2^ Department of Emergency Medicine, Shahid Sadoughi University of Medical Sciences, Yazd, Iran; ^3^ Department of General Surgery, Shahid Sadoughi University of Medical Sciences, Yazd, Iran; ^4^ Department of Forensic Medicine, Shahid Beheshti University of Medical Sciences, Tehran, Iran; ^5^ Department of Pediatric Dentistry, Arak University of Medical Sciences, Arak, Iran; ^6^ Department of Endodontic, Arak University of Medical Sciences, Arak, Iran; ^7^ Department of Restorative and Esthetic, Arak University of Medical Sciences, Arak, Iran; ^8^ Mother and Newborn Health Research Center, Shahid Sadoughi University of Medical Sciences, Yazd, Iran

**Keywords:** Breast cancer, *SULT1A1 gene*, Association, Meta-analysis

## Abstract

**Background:** The *Arg213His* (rs9282861) polymorphism of Sulfotransferase Family 1A Member 1 (*SULT1A1*) gene has been reported to be associated with risk of breast cancer in some epidemiological studies. However, the results of these studies are conflicting and inconclusive. Therefore, this systematic review and meta-analysis was conducted to evaluate the association of *SULT1A1 Arg213His* (rs9282861) polymorphism with susceptibility to breast cancer.

**Study design:** A systematic review and meta-analysis.

**Methods:** A comprehensive literature search for eligible studies was conducted in PubMed, Elsevier, Science Direct, Scopus and Google Scholar databases up to October 5, 2017. Pooled odds ratios (ORs) with their corresponding 95% confidence intervals (95% CIs) were used to evaluate the strength of the association using fixed effects models and random effects models.

**Results:** A total of 20 relevant case-control studies involving 11,077 cases and 14,798 controls were included in this meta-analysis. Overall, there was a significant association between the *SULT1A1 Arg213His* (rs9282861) polymorphism and risk of breast cancer in the allele mode (A vs. G: OR=1.117, 95% CI: 1.011, 1.233, *P*=0.029) and the homozygote model (AA vs. GG: OR=1.288, 95% CI: 1.036, 1.601, *P*=0.022). Subgroup analysis based on ethnicity suggested *SULT1A1 Arg213His* (rs9282861) polymorphism had a subtly increased breast cancer risk among Asian population, but not Caucasians. Further, subgroup analyses, significant associations were observed in hospital based group, RFLP-PCR group, and high quality studies subgroups.

**Conclusions:** This meta-analysis suggested that *SULT1A1 Arg213His* (rs9282861) polymorphism might be associated with breast cancer risk, especially among Asian population. Moreover, the *SULT1A1 Arg213His* polymorphism is of high clinical relevance by ethnicity and would be a useful marker to identify patients who are at higher risk for breast cancer.

## Introduction

Breast cancer is one of the most common malignancies among women worldwide ^1,2^. Currently, more women survive due to earlier diagnosis and better therapy ^3,4^. However, the morbidity and mortality of breast cancer have increased in most developing countries^5^. It is well-established that family or personal history of cancer, nulliparous, and history of hormone replacement therapy and different genetic background are most known risk factors for breast cancer ^6^. Breast cancer is one of the first multifactorial conditions to be evaluated using molecular genetics techniques ^6-8^. It is estimated that 15- 30% of hereditary breast cancer cases are due to known highly penetrant genes such as BRCA1 and BRCA2 9. More than 1,000 different breast cancer-associated mutations in each gene have been documented. However, a few of these mutations could be clearly identified as pathological ^9-11^.

Genetic variations in *SULT1A1* gene are increasingly studied for an increased different cancer risk because of the critical roles in catalyzing the sulfate conjugation of many hormones, neurotransmitters, drugs, and xenobiotic compounds ^12^. In particular, *SULT1A1* has high activity towards a wide range of substrates including environmental carcinogens such as tobacco. The *SULT1A1* gene is located on chromosome 16p12.1-p11.2, spans approximately 5.1 kb and contains nine exons that range in length from 74 to 347 bp ^13^. To date, the Arg213His (rs9282861) polymorphism is most widely studied SNP within *SULT1A1* gene. The Arg213His polymorphism is located in exon 7 with G to A transition, at nucleotide 638 (codon 213) leading to an arginine (Arg) to histidine (His) amino acid substitution ^14^. Functional studies have reported that the variant A allele (His allele) is associated with lower sulfotransferase activity and thermal stability compared with the wild-type G allele (Arg allele) ^15,16^.

So far, there were so many reports about the association of *SULT1A1* Arg213His (rs9282861) polymorphism with breast cancer risk ^17-35^. On the whole, these studies results were conflicting and inconclusive, probably due to the relatively small size, ethnic background, uncorrected multiple hypothesis testing, and publication bias. It is known that meta-analysis is a statistical procedure for combining data from multiple studies to produce a single estimate on the same outcome of interest with enhanced evaluation. Therefore, current systematic review and meta-analysis was performed from all eligible studies to derive a more precise estimation the association between *SULT1A1* Arg213His (rs9282861) polymorphism and breast cancer risk.

## Methods

### Search Strategy

We performed a systematic literature search of electronic databases such as PubMed, Embase, ISI Wed of Knowledge, Google scholar, Scopus, Cochrane Library, Chinese National Knowledge Infrastructure (CNKI) and Chinese Wan Fang databases to identify studies that evaluated the association between *SULT1A1 Arg213His* (rs9282861) polymorphism and the risk of breast cancer up to October 5, 2017. The search strategy identified all possible studies using combinations of the following keywords: Sulfotransferase Family 1A Member, *SULT1A1* gene, G638A, Arg213His, rs9282861, and polymorphism, genetic polymorphism, single nucleotide polymorphism, SNP mutation, and breast, breast cancer, breast malignancy, breast tumor, breast carcinoma, and breast neoplasm. In addition, manual searching was carried out to ensure that no relevant studies were missed in the database search by scanning the review articles, clinical trials, and meta-analyses. No language restrictions were imposed.

### Inclusion and Exclusion Criteria

To be eligible for the inclusion criteria in the meta-analysis, the following criteria were used: a) case-control or cohort design; b) evaluating the associations between *SULT1A1 Arg213His* (rs9282861) polymorphism and breast cancer risk; c) providing sufficient data to estimate the odds ratio (OR) with the corresponding 95% confidence interval (95%CI). Studies were excluded if the following criteria were satisfied: a) Abstracts, case reports, case-only articles, family-based studies, reviews articles and repeated literatures; b) duplicate of previous publications; c) not provided sufficient data (e.g. neither the frequency nor the number of genotype was given) for calculation.

### Data Extraction

All data were extracted independently by two authors using a standardized data extraction form and according to the inclusion criteria listed above. The following elements were extracted for each included study: first author name, year of publication, country, ethnicity, source of control groups, genotyping method, total number of cases and controls, minor allele frequency (MAF) in control subjects, *P* value of the Hardy–Weinberg equilibrium (HWE) test in the control. Different ethnic descents were classified as Caucasian, Asian, Latinos or Mixed (derived from an admixture of different ethnic groups). Disagreements between the two investigators were resolved by discussing with a third investigator. If more than one article were published using the same data, only the newest study and with largest sample size was selected. Moreover, the quality of selected studies was tested by the confirmation of HWE in control groups, and studies without the confirmation of HWE in controls were defined as low-quality studies, while studies with the confirmation of HWE in controls were defined as high-quality studies.

### Statistical Analysis

Crude odds ratios (ORs) with corresponding 95% confidence intervals (CIs) were used to assess the strength of association between the *SULT1A1 Arg213His* (rs9282861) polymorphism and breast cancer risk. The pooled ORs were determined for *SULT1A1 Arg213His* (rs9282861) polymorphism under the allele model (A vs. G), the homozygote model (AA vs. GG), the heterozygote model (AG vs. GG), the dominant model (AA+AG vs. GG), and the recessive model (AA vs. AG+GG). The significance of the pooled OR was determined by the Z-test, in which *P*<0.05 was considered as statistically significant. The Chi-square-based Q-test and I2-statistics were employed for evaluating the between-study heterogeneity36,37. A *P*-value greater than 0.10 for the Q-test or I2 less than 50% indicates a lack of heterogeneity among studies, so the pooled OR estimate of the included studies was obtained by the fixed-effects model (Mantel-Haenszel method) 38. Otherwise, the random-effects model (DerSimonian-Laird method) was used 39. Hardy-Weinberg equilibrium (HWE) in the control group was calculated by the Chi-square test for goodness of fit, If *P* value > 0.05, the genotype distribution of control population conformed to HWE. Furthermore, to explore the sources of heterogeneity, subgroup analysis we conducted based on ethnicity, source of controls and studies quality. To validate the reliability of the results, sensitivity analysis was performed though omitting one case-control study each time, as well as limiting this meta-analysis to studies which were conformed to HWE. Publication bias was evaluated by Begg’s test and Egger’s test (*P* <0.05 was considered statistically significant)40,41. If publication bias existed, the Duval and Tweedie non-parametric ‘‘trim and fill’’ method was used to adjust for it. All statistical analyses including meta-regression, sensitivity analysis, subgroup analysis, and publication bias assessment were performed using Comprehensive Meta-Analysis (CMA) software version 2.0 (Biostat, USA). Two-sided *P* values < 0.05 were considered statistically significant.

## Results

### Characteristics of Studies

**Figure 1:**
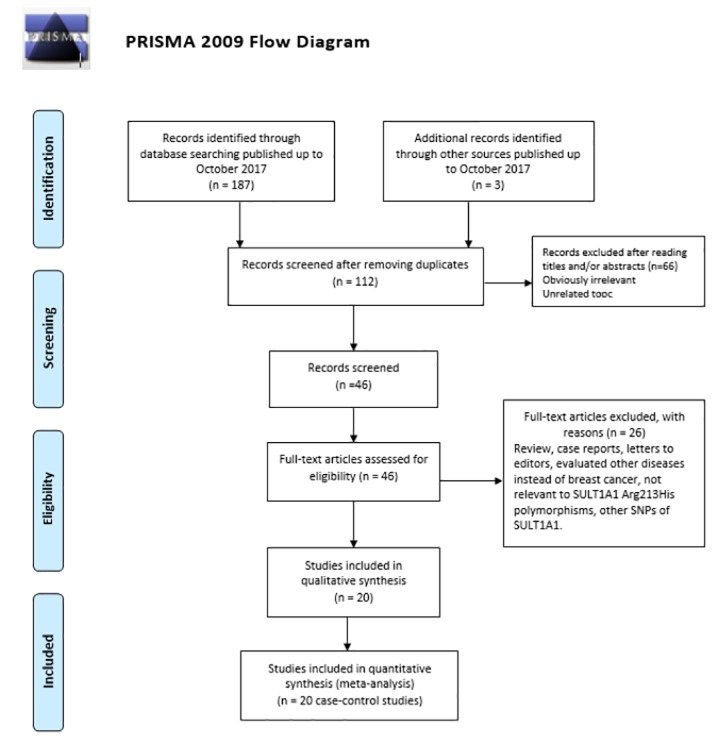
Flow chart of literature search and study selection

As shown in Figure 1, we initially identified 187 potentially relevant studies from database searching and 3 studies from manual retrieval. After screening of titles or abstracts duplicates were excluded resulting in 112 publications. An additional 92 publications were excluded because the studies were reviews, case reports, letter to editor, not reporting the usable data, other polymorphisms of *SULT1A1* gene, not related to breast cancer or reported other diseases. Finally, a total of 20 studies with 11,077 cancer cases and 14,798 controls met the inclusion criteria. Study characteristics are summarized in Table 1. The included studies were published from 2000 to 2013. Of those studies, there were twelve studies of Caucasian descendants and eight studies of Asian descendants. The countries of these studies were USA, Austria, India, Korea, Sweden, Germany, Finland, China, Taiwan, Russia, and Italy. All the 20 eligible studies were case-control studies, 11 of them were in a population-based design and 8 studies were hospital based. The genotyping for *SULT1A1 Arg213His* (rs9282861) polymorphism was performed by five genotyping methods including real-time PCR (RT-PCR), PCR-restriction fragment length polymorphism (PCR-RFLP), TaqMan, Genotyping by matrix-assisted laser desorption/ionization time-of-flight mass spectrometry (MALDI-TOF MS) and direct Sequencing. The distribution of genotype in the controls of the studies was not in agreement with Hardy-Weinberg equilibrium in six studies, which were further tested in the sensitivity analyses and subgroup analysis of studies with high quality. The detailed characteristics of each study and *SULT1A1 Arg213His* (rs9282861) polymorphism genotype distributions included in the meta-analysis are presented in Table 1.

**Table 1:** Main characteristics of studies included in this meta-analysis

**First author/Year**	**Country ethnicity**	**SOP**	**Genotyping** **method**	**Case**	**Control**	**Cases**					**Controls**					**MAFs**	**HWE**
						**Genotype**			**Allele**		**Genotype**			**Allele**			
						**GG**	**AG**	**AA**	**G**	**A**	**GG**	**AG**	**AA**	**G**	**A**		
Seth 2000 17	USA(Caucasian)	HB	RT-PCR	444	227	229	176	39	634	254	110	94	23	314	140	0.308	0.193
Zheng 2001 18	USA(Caucasian)	PB	PCR-RFLP	155	326	55	71	29	181	129	147	135	44	429	223	0.342	0.148
Tang 2003 19	USA(Caucasian)	HB	PCR-RFLP	103	133	50	42	11	142	64	79	47	7	205	61	0.229	0.997
Langsenlehner 2004 20	Austria (Caucasian)	PB	TaqMan	498	499	201	250	47	652	344	224	212	63	660	338	0.338	0.249
Chacko 2004 21	India(Asian)	HB	PCR-RFLP	140	140	76	56	8	208	72	95	41	4	231	49	0.175	0.866
Choi 2005 22	Korea(Asian)	Mixed	TaqMan	986	1045	796	190	0	1782	190	830	215	0	1875	215	0.102	0.001
Jerevall 2005 23	Sweden(Caucasian)	PB	PCR-RFLP	229	228	80	121	28	280	178	84	106	38	274	182	0.399	0.642
Le Marchand 2005 24	USA(Caucasian)	PB	PCR-RFLP	1339	1370	801	424	114	2026	652	782	484	104	2048	692	0.252	0.017
Lilla 2005 25	Germany(Caucasian)	PB	PCR-RFLP	419	884	198	169	52	565	273	374	403	107	1151	617	0.349	0.922
Sillanpaa 2005 26	Finland(Caucasian)	PB	PCR-RFLP	480	478	145	229	106	519	441	147	221	110	515	441	0.461	0.127
Yang 2005 27	China(Asian)	PB	MALDI-TOF	1102	1147	921	181	0	2023	181	977	170	0	2124	170	0.074	0.006
Cheng 2005 28	Taiwan(Asian)	HB	PCR-RFLP	468	740	439	27	2	905	31	693	47	0	1433	47	0.031	0.372
Han 2005 29	China(Asian)	HB	PCR-RFLP	209	426	160	41	8	364	54	355	61	10	771	81	0.095	0.001
Gulyaeva 2008 30	Russia(Caucasian)	PB	PCR-RFLP	82	180	23	40	19	99	65	63	61	56	187	173	0.480	0.001
Syamala 2010 31	India(Asian)	HB	PCR-RFLP	359	367	244	87	18	591	127	271	90	6	632	102	0.139	0.635
Merie-Genica 2010 32	Germany(Caucasian)	PB	MALDI-TOF	3139	5426	1381	1332	426	4094	2184	2338	2430	658	7106	3746	0.345	0.491
Serrano 2011 33	Italy(Caucasian)	HB	RT-PCR	46	136	24	18	4	66	26	71	55	10	197	75	0.275	0.883
Khvostova 2012 34	Russia(Caucasian)	PB	PCR-RFLP	335	530	47	164	103	275	395	166	261	103	593	467	0.440	0.982
Lee 2012 35	China (Asian)	PB	Sequencing	400	400	300	83	17	683	117	310	78	12	698	102	0.127	0.013
Kumar 2013 42	India(Asian)	HB	PCR-RFLP	144	116	117	25	2	259	29	111	5	0	227	5	0.021	0.812

SOP, source of population; HB, Hospital-based study; PB, Population-based study; RT-PCR, Real-Time PCR; PCR-RFLP, PCR-restriction fragment length polymorphism; MALDI-TOF MS, matrix-assisted laser desorption/ionization time-of-flight mass spectrometry (MALDI-TOF MS); MAFs, Minor Allele Frequencies; HWE, Hardy–Weinberg equilibrium.

### Quantitative Synthesis

Table 2 listed the main results of the meta-analysis of *SULT1A1 Arg213His* (rs9282861) polymorphism and breast cancer risk. The random effects model was chosen to synthesize the data from the allelic model, homozygote, heterozygote, dominant model and recessive model. Overall, there was a significant association between *SULT1A1 Arg213His* (rs9282861) polymorphism and breast cancer in the allele mode (A vs. G: OR=1.117, 95% CI: 1.011, 1.233, *P*=0.029) and the homozygote model (AA vs. GG: OR=1.288, 95% CI: 1.036, 1.601, *P*= 0.022), but not in the heterozygote model (AG vs. GG: OR = 0.854, 95% CI 0.705-1.036, *P*= 0.109), the dominant model (AA+AG vs. GG: OR=1.109, 95% CI: 0.990, 1.241, *P*= 0.073), and the recessive model (AA vs. AG+GG: OR=1.144, 95% CI: 0.969, 1.351, *P*= 0.112).

In the subgroup analysis by the ethnicity, a significant association between the *SULT1A1 Arg213His* (rs9282861) polymorphism and breast cancer was observed in Asians in the allele model (A vs. G: OR=1.117, 95% CI: 1.011, 1.233, *P*= 0.020), the homozygote model (AA vs. GG: OR=2.112, 95% CI: 1.342, 3.323, *P*= 0.001), the heterozygote model (AG vs. GG: OR=0.468, 95% CI: 0.249, 0.881, P=0.019), the dominant model (AA+AG vs. GG: OR=1.237, 95% CI: 1.008, 1.518, *P*=0.041) and the recessive model (AA vs. AG+GG: OR=1.990, 95% CI: 1.268, 3.125, *P*=0.003), but not in the Caucasian populations.

Moreover, the studies were stratified on the basis of genotyping method, source of control subjects and high quality studies (HWE status). In the PCR-RFLP group, significantly increased association between *SULT1A1 Arg213His* polymorphism and risk of breast cancer were found in the allele mode (A vs. G: OR=1.209, 95% CI: 1.019, 1.435, *P*=0.030), the homozygote model (AA vs. GG: OR=1.504, 95% CI: 1.081, 2.093, *P*=0.015), and in the heterozygote model (AG vs. GG: OR=0.753, 95% CI: 0.570, 0.993, *P*=0.045). Interestingly, the stratifying by source of population showed that *SULT1A1 Arg213His* (rs9282861) polymorphism was significantly associated with breast cancer risk in the allele mode (A vs. G: OR=1.319, 95% CI: 1.044, 1.666, *P*=0.020) and the homozygote model (AA vs. GG: OR=1.523, 95% CI: 1.067, 2.175, *P*=0.021), the dominant model (AA+AG vs. GG: OR=1.319, 95% CI: 1.013, 1.717, *P*=0.040), and the recessive model (AA vs. AG+GG: OR=1.459, 95% CI:1.030, 2.066, *P*=0.033) in the hospital based group, but not population based (Table 2). Subgroup analysis of studies with high quality showed that there was a significant association between *SULT1A1 Arg213His* (rs9282861) polymorphism and increased risk of breast cancer in the allele model (OR=1.174, 95% CI: 1.026, 1.343, *P*=0.019), the homozygote model (OR=1.343, 95% CI: 1.015, 1.777, *P*=0.039) and the dominant model (AA+AG vs. GG: OR=1.175, 95% CI: 1.005, 1.373, *P*=0.043, Table 2).

**Table 2:** The meta-analysis of *SULT1A1 Arg213His* (rs9282861) polymorphism and breast cancer risk

**Subgroup**	**Study No.**	**Genetic Model**	**Type of Model**	**Heterogeneity**			**Odds Ratio**			**Publication Bias**	
				**I2 (%)**	**PH**	**OR**	**95% CI**	**Ztest**	**POR**	**PBeggs**	**PEggers**
**Overall**	20	A vs. G	Random	73.77	0.001	1.117	1.011, 1.233	2.184	0.029	0.029	0.077
	18	AA vs. GG	Random	65.91	0.001	1.288	1.036, 1.601	2.283	0.022	0.111	0.129
	18	AG vs. GG	Random	53.88	0.004	0.854	0.705, 1.036	-1.604	0.109	0.225	0.178
	18	AA+AG vs. GG	Random	66.10	0.001	1.109	0.990, 1.241	1.792	0.073	0.047	0.007
	18	AA vs. AG+GG	Random	50.44	0.008	1.144	0.969, 1.351	1.589	0.112	0.100	0.284
**By Ethnicity**											
**Caucasian**	12	A vs. G	Random	77.83	0.001	1.061	0.942, 1.194	0.971	0.331	0.303	0.589
	12	AA vs. GG	Random	72.47	0.001	1.167	0.925, 1.473	1.302	0.193	0.945	0.665
	12	AG vs. GG	Random	58.37	0.006	0.915	0.755, 1.108	-0.913	0.361	0.945	0.833
	12	AA+AG vs. GG	Random	65.76	0.001	1.045	0.914, 1.196	0.648	0.517	0.195	0.372
	12	AA vs. AG+GG	Random	56.35	0.009	1.067	0.900, 1.264	0.745	0.456	0.731	0.619
**Asian**	8	A vs. G	Random	62.04	0.010	1.251	1.037, 1.509	2.335	0.020	0.063	0.011
	6	AA vs. GG	Fixed	0.00	0.732	2.112	1.342, 3.323	3.231	0.001	0.452	0.144
	6	AG vs. GG	Fixed	16.69	0.306	0.468	0.249, 0.881	-2.353	0.019	0.707	0.253
	6	AA+AG vs. GG	Random	61.49	0.011	1.237	1.008, 1.518	2.039	0.041	0.035	0.012
	6	AA vs. AG+GG	Fixed	0.00	0.724	1.990	1.268, 3.125	2.990	0.003	0.452	0.146
**By Genotyping Method **										
**PCR-RFLP**	13	A vs. G	Random	81.51	0.001	1.209	1.019, 1.435	2.174	0.030	0.058	0.108
	13	AA vs. GG	Random	71.98	0.001	1.504	1.081, 2.093	2.423	0.015	0.360	0.148
	13	AG vs. GG	Random	56.23	0.007	0.753	0.570, 0.993	-2.007	0.045	0.246	0.045
	13	AA+AG vs. GG	Random	73.95	0.001	1.207	0.995, 1.464	1.912	0.056	0.127	0.026
	13	AA vs. AG+GG	Random	56.18	0.007	1.249	0.983, 1.586	1.822	0.068	0.360	0.208
**Source of Population **										
**Hospital Based**	8	A vs. G	Random	63.32	0.008	1.319	1.044, 1.666	2.322	0.020	0.265	0.062
	8	AA vs. GG	Fixed	33.80	0.158	1.523	1.067, 2.175	2.316	0.021	1.000	0.058
	8	AG vs. GG	Fixed	31.81	0.174	0.694	0.470, 1.024	-1.842	0.066	0.901	0.069
	8	AA+AG vs. GG	Random	60.17	0.014	1.319	1.013, 1.717	2.058	0.040	0.173	0.074
	8	AA vs. AG+GG	Fixed	23.15	0.245	1.459	1.030, 2.066	2.128	0.033	0.536	0.044
**Population Based**	11	A vs. G	Random	78.40	0.001	1.070	0.953, 1.201	1.144	0.253	0.350	0.543
	10	AA vs. GG	Random	75.54	0.001	1.181	0.925, 1.509	1.337	0.181	0.858	0.671
	11	AG vs. GG	Random	64.39	0.003	0.909	0.740, 1.117	-0.906	0.365	1.000	0.832
	11	AA+AG vs. GG	Random	67.87	0.001	1.058	0.929, 1.203	0.850	0.395	0.350	0.165
	10	AA vs. AG+GG	Random	60.88	0.006	1.073	0.898, 1.281	0.778	0.437	0.591	0.547
**High Quality Studies ( HWE)**										
	14	A vs. G	Random	78.55	0.001	1.174	1.026, 1.343	2.340	0.019	0.021	0.083
	14	AA vs. GG	Random	72.94	0.001	1.343	1.015, 1.777	2.064	0.039	0.511	0.187
	14	AG vs. GG	Random	46.92	0.027	0.904	0.746, 1.095	-1.034	0.301	0.912	0.754
	14	AA+AG vs. GG	Random	70.51	0.001	1.175	1.005, 1.373	2.026	0.043	0.062	0.009
	14	AA vs. AG+GG	Random	57.15	0.004	1.168	0.953, 1.432	1.496	0.135	0.381	0.346

PCR-RFLP, PCR-restriction fragment length polymorphism; HWE, Hardy–Weinberg equilibrium.

### Minor allele frequencies (MAFs)

The MAFs for the *SULT1A1 Arg213His* (rs9282861) polymorphism in the healthy controls is presented in Table 1. The allele and genotype distributions of *SULT1A1 Arg213His* (rs9282861) polymorphism exhibited ethnic variations. The allele and genotype distributions of 213His allele in the Caucasian and Asians populations were 32.75% (17.5%-48.0%) and 7.4% (2.1%-12.7%), respectively. Thus, the frequency of the 213His allele in Asians was less than Caucasians.

### Heterogeneity analysis

There was a significant heterogeneity in overall comparisons under all genetic models among the overall 20 studies of the *SULT1A1 Arg213His* (rs9282861) polymorphism (e.g., allele model: Q= 72.44 on 19 d.f (Q), *P*= ≤0.001, I2 = 73.77%; and dominant model: Q= 189.01 on 17 d.f (Q), *P*= ≤0.001, I2 = 91%). Therefore, we have performed subgroup analyses by ethnicity and HWE status to explore the source of heterogeneity. The I2 decreased obviously and *P* value exceeded 0.05 under the homozygote, heterozygote and recessive models among Asians, while it was still significant under all genetic models among Caucasians (Table 2). The results indicated that ethnicity may be a source of heterogeneity in the current meta-analysis.

### Test of heterogeneity and sensitivity analyses

There was significant heterogeneity among these studies for *SULT1A1 Arg213His* (rs9282861) polymorphism in the allele mode (A vs. G: *P*<0.001), the homozygote model (AA vs. GG: *P*<0.001), the heterozygote model (AG vs. GG: *P*=0.004), the dominant model (AA+AG vs. GG: *P*<0.001), and the recessive model (AA vs. AG+GG: *P*=0.008). Then, the source of heterogeneity was assessed by meta-regression analysis. However, the ethnicity, genotyping methods, studies quality and source of population did not contribute to substantial heterogeneity in this meta-analysis (Table 2). In addition, the sensitivity analysis was performed to assess the reliability and conclusiveness of the overall results by repeating the meta-analysis sequentially removing each individual study. However, the results showed that the pooled ORs were not considerably affected by removing any individual study in all five genetic models, which indicated the reliability of our results.

### Publication Bias

**Figure 2:**
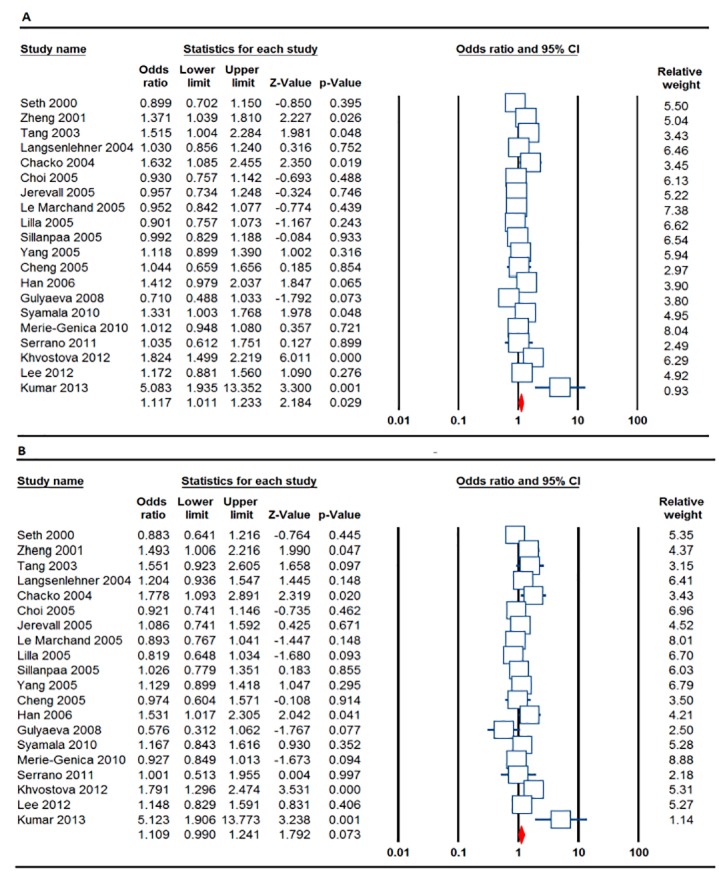
Forest plot of the association between *SULT1A1 Arg213His* (rs9282861) polymorphism and breast cancer disease. “A” represents allele model (A vs. G); and “B” represents the dominant model (AA+AG vs. GG). Every study was represented by a square whose size was proportional to the weight of the study. Diamond indicated summary odds ratios (OR) with its corresponding the pseudo 95% confidence limits (95% CI)

**Figure 3:**
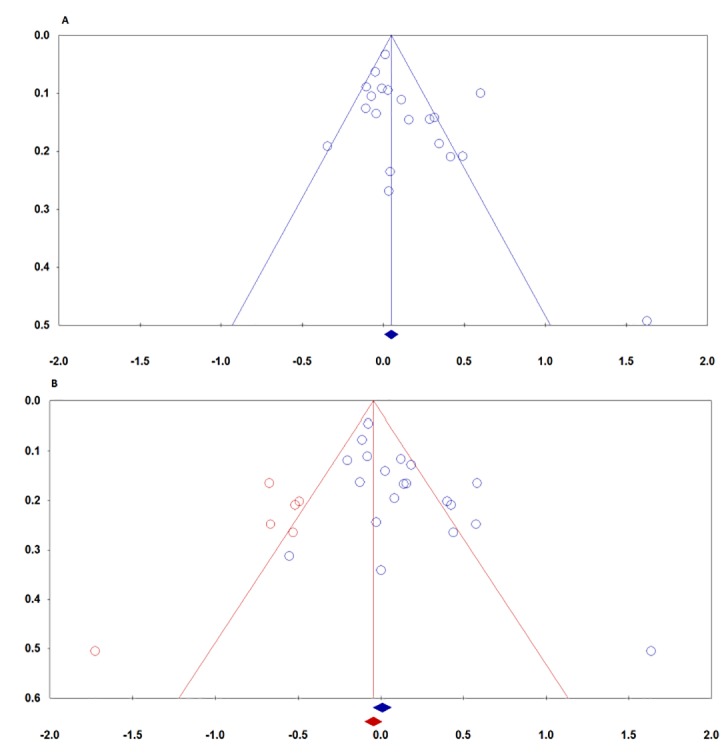
Begg’s funnel plots of publication bias test for the association between the SULT1A1 Arg213His (rs9282861) polymorphism and breast cancer risk. “A” represents the allele model (A versus G); and “B” represents the dominant model (AA+AG vs. GG, with trim and fill test). The dots represent specific studies for the indicated association.

We have used both Begg’s funnel plot and Egger’s test to access the small study effects of the literatures. The visual inspection of funnel plot revealed no obvious asymmetry in the allele (Figure 2A), homozygote, heterozygote and recessive models. However, the results of Egger’s test showed a publication bias for association of *SULT1A1 Arg213His* (rs9282861) polymorphism with risk of breast cancer in dominant model (AA+AG vs. GG: *P*_Begg_=0.047, *P*_Egger_=0.007, Figure 2B). Therefore, the Duval and Tweedie non-parametric ‘‘trim and fill’’ method was used to adjust for publication bias. Meta-analysis with and without ‘‘trim and fill’’ did not draw different conclusion, indicating that the results were statistically robust (Fig. 3B).

## Discussion

The pathogenesis of breast cancer is complex and genetic factor play an important role in breast cancer susceptibility. An increasing number of publications on genetic association studies, genome-wide association studies (GWASs), and relate meta-analyses have been published to clarify the association between different gene polymorphisms and breast cancer. To our knowledge, this is the most comprehensive and robust meta-analysis to explore and report the association between the *SULT1A1 Arg213His* (rs9282861) polymorphism and breast cancer. The pooled ORs of 20 case-control studies with ^11^,077 cases and ^14^,798 controls in the current meta-analysis concluded that there was a significant correlation between *SULT1A1 Arg213His* (rs9282861) polymorphism and breast cancer under allele mode (A vs. G: OR=1.117, 95% CI: 1.011-1.233, *P*=0.029) and homozygote model (AA vs. GG: OR=1.288, 95% CI: 1.036, 1.601, *P*=0.022).

Our results are inconsistent with the previous meta-analysis by Xiao et al., which not reported a significant association between *SULT1A1 Arg213His* polymorphism and breast cancer risk ^12^. The current meta-analysis is different from previous meta-analysis. We collected all 20 eligible studies, which include 13 studies used by Xiao et al., researches and seven new cases-control studies. It was remarkable that they have not included the Choi et al. 2003 22, Le Marchand et al. 2005 ^24^, Yang et al. 2005 ^27^, Han et al. 2006^29^, and Gulyaeva et al. 2008 ^30^ studies with that published up to their electronic researches due to lack of agreement with HWE. We analyzed the association between *SULT1A1 Arg213His* (rs9282861) polymorphism and breast cancer in Caucasian and Asian populations. The association is significant in Asians under in the allele model (A vs. G: OR=1.117, 95% CI: 1.011, 1.233, *P*=0.020), the homozygote model (AA vs. GG: OR=2.112, 95% CI: 1.342, 3.323, *P*=0.001), the heterozygote model (AG vs. GG: OR=0.468, 95% CI: 0.249, 0.881, *P*=0.019), the dominant model (AA+AG vs. GG: OR=1.237, 95% CI: 1.008, 1.518, *P*=0.041) and the recessive model (AA vs. AG+GG: OR=1.990, 95% CI: 1.268, 3.125, *P*=0.003), but not among Caucasian populations. Our findings suggested that *SULT1A1 Arg213His* (rs9282861) polymorphism might have a susceptible nature in the Asians, but not in the Caucasians. Therefore, different populations lived in different environments and gene-environment interactions may partly affect the breast cancer susceptibility. Compared with the current meta-analysis, Xiao et al. not performed subgroup analysis by ethnicity for breast cancer ^12^. Therefore, Xiao et al., study essentially remains an open field as meta-analysis their results reliability due lack of subgroup analysis by ethnicity and the number of studies were considerably smaller than that needed to receive the robust conclusions.

Interestingly, stratified analysis according to genotyping method showed a significant association between *SULT1A1 Arg213His* polymorphism and increased risk of breast cancer in participants of those studies involving PCR-RFLP under the allele mode (A vs. G: OR=1.209, 95% CI: 1.019, 1.435, *P*=0.030), the homozygote model (AA vs. GG: OR=1.504, 95% CI:1.081, 2.093, *P*=0.015), and in the heterozygote model (AG vs. GG: OR=0.753, 95% CI: 0.570, 0.993, *P*=0.045). We suggested that this trend is possible for that most of the included studies mainly utilized PCR-RFLP method to genotyping. Moreover, this result should be carefully interpreted and confirmed by conducting further analysis of additional published studies.

Between-study heterogeneity might distort the conclusion of a meta-analysis ^43,44^. The study designs, source of controls subjects, ethnicity, genotyping method, sample size, lifestyle, subject’s age and gender may contribute to the heterogeneity ^45^. In the present meta-analysis, significant between-study heterogeneity was detected across studies under all models; therefore, we utilized the random-effects model to summarize the ORs. Additionally, in order to make the conclusion more credible, we performed the publication bias analysis and sensitivity analysis. Funnel plots suggested that no obvious publication bias was observed. Xiao et al. ^12^ removed the study conducted by Khvostova et al. ^34^ due to the low-quality and results indicated that this study influences the pooled estimates and the heterogeneity most in breast cancer subgroup. We have also removed Khvostova et al. ^34^ study, but there was no obvious effect on the pooled ORs and the corresponding CIs. In addition, we have performed the sensitivity analysis by removing those studies departed from HWE, but the results did not change meaningfully by excluding six studies departed from HWE or one study with low-quality. We suggest the relative small sample size status may be the reason for Xiao et al. ^12^ observations.

Despite the advantage of large sample size and no evidence of publication bias, the meta-analysis had several limitations that should be taken into account. First, the population data of *SULT1A1 Arg213His* (rs9282861) polymorphism are limited for the subgroup analysis, since there were not enough studies in other population or regions such as Africans and Middle East in this meta-analysis. Therefore there was insufficient statistical power to demonstrate the associations between the * SULT1A1 Arg213His* (rs9282861) polymorphism and breast cancer risk in Africans or Middle East. Second, we only included articles that were published in English, and language bias might exist. Third, because of moderate to high heterogeneity in all five genetic models, we used the random effect model, which is not as reliable as the fixed-effects model and may potentially restrict the interpretation of the pooled risk estimates. Although the degree of heterogeneity was reduced (but not disappeared) by subgroup analyses based on ethnicity and HWE status, other sources of heterogeneity were not checked. Forth, in the current meta-analysis the design of all included studies was case control, therefore results more prone to selection bias. Fifth, the current meta-analysis results were based on unadjusted estimates, while a more precise analysis should be performed if all individual data available, which would allow for the adjustment by other co-variants including age, environmental exposures, smoking status, reproductive history, menopausal status and other lifestyle factors. Finally, gene-gene and gene-environment interactions which may modulate the breast cancer susceptibility were limited owing to the lack of the sufficient data in the eligible studies.

In summary, despite the limitations, this study is the most comprehensive, best available evidence and accurate meta-analysis focusing on the association between *SULT1A1 Arg213His* (rs9282861) polymorphism and risk of breast cancer. The present meta-analysis provided evidence of association between *SULT1A1 Arg213His* (rs9282861) polymorphism and increased breast cancer risk, especially among Asians. These results suggest that the *SULT1A1 Arg213His* (rs9282861) polymorphism is of high clinical relevance among Asians and would be a useful marker to identify women who are at higher risk for breast cancer. Moreover, well-designed epidemiological studies with larger sample size, carefully matching cases and control subjects and more ethnic groups are needed to validate these results. Further studies may focus on the influence of gene-gene and gene-environment interactions on the association of *SULT1A1 Arg213His* (rs9282861) polymorphism and breast cancer.

## Acknowledgements

We thank Prof. Seyed Mehdi Kalantar and Prof. Mohammad Hassan Sheikhha for their great support acknowledged.

## Conflict of interest statement

The authors declared no conflict of interest.

## Funding

This research received no specific grant from any funding agency in the institute or university.

## Highlights

- The * SULT1A1 Arg213His* polymorphism is associated with increased risk of breast cancer.
- The * SULT1A1 Arg213His* polymorphism may be associated with increased risk of breast cancer in **Asians**.
- The * SULT1A1 Arg213His* polymorphism would be a useful marker to identify women who are at higher risk of breast cancer.
